# Safeguarding Data Integrity in Online Podiatry Research: Understanding and Managing Non‐Genuine Participation

**DOI:** 10.1002/jfa2.70141

**Published:** 2026-02-28

**Authors:** Will Gurr, Michael McDougall, Abigail O’Brien, Kate Carter

**Affiliations:** ^1^ Department of Podiatric Medicine and Surgery School of Health and Clinical Sciences The University of Western Australia Perth Western Australia Australia

## Abstract

Online research methods have become increasingly common in podiatry, offering efficient, low cost and convenient data collection. Emerging evidence from online studies suggests that the integrity of online research is being compromised by participants who are not genuine: for example, they may not have the relevant health condition they are claiming to have or they may not be taking the trial medication as instructed. In health research, the consequences of non‐genuine participants are significant, including unreliable data, wasted time and funding, researcher distress, loss of participant trust and, in some cases, cancelled projects. Growing awareness of fraudulent participation and reports of these challenges highlights the need for structured approaches designed to safeguard data integrity and the trustworthiness of results. In the United Kingdom, the University of Nottingham has recently published practical guidance for researchers and ethics committees on handling potential non‐genuine participants. The aim of this commentary is to raise awareness of the risks posed by non‐genuine participation in online research and to provide a summary of the recently published practical strategies for researchers to safeguard the integrity of their data.

## 1 Background

While online research methods are used increasingly to allow recruitment of large samples, ensuring anonymity and providing convenience for both researchers and participants [1], these same advantages create vulnerabilities through the lack of in‐person validation. It is now widely acknowledged that non‐genuine participation affects most forms of online research, including interviews, surveys and clinical recruitment [2, 3, 4, 5, 6, 7]. Non‐genuine participation has been defined into five categories [3, 8]: (1) inauthentic participants faking their lived experience, (2) repeat responders taking part more than once, (3) misrepresentation of eligibility including exaggeration or distortion of specific details, (4) disengaged responders answering quickly giving careless responses and not paying attention and (5) automated bot activity completing surveys or research tasks simulating human participation. Despite use of strict inclusion/exclusion criteria and the development of advanced secure platforms, all these categories persist to affect both quantitative and qualitative web‐based data collection [3].

In several documented health studies, over half the participant submissions were suspected to be non‐genuine [3]. Published personal experiences of researchers include interviewing people pretending to have a lived experience of attention‐deficit/hyperactivity disorder in an online study [3, 9], with 66% (8/12) of participants found to be non‐genuine, it was not possible to complete or publish the project as participants were paid $20 per interview and there was no extra budget or time available to conduct additional interviews. Other reports included individuals creating multiple identities in a randomised controlled trial with 99% (482/483) suspected as non‐genuine participants [3]. Non‐genuine participation may occur by accident from misunderstandings, or deliberately to gain financial incentives, early access to interventions, or to attempt to influence outcomes [2]. Irrespective of the cause, the negative impact on the reliability of findings, wasted resources and additional ethical and administrative burdens on research teams can be significant [3]. Furthermore, genuine participants may be denied reimbursement or unfairly questioned, potentially causing a loss of trust in the project and research in general. Perhaps most importantly, flawed data could misinform evidence‐based practice and lead to ineffective or unsafe clinical recommendations [10].

In podiatry, use of reporting guidelines for survey and online research is common practice [11, 12, 13] as well as the handling of missing data. Previously underreported in online survey research [14], transparent reporting of missing data is the recommended standard of good practice [15]. Despite the widespread recognition of non‐genuine participation [16, 17, 18, 19, 20, 21, 22], few studies in podiatry have reported on rigorous methodologies to prevent, detect and report this problem. Addressing these risks requires careful planning, early intervention and ongoing vigilance, with strategies tailored to different study contexts and participant populations [6]. Protections must also be proportionate so as not to disadvantage or deter genuine participants [8]. The 2025 University of Nottingham guidance provides a timely, structured framework to recognise, mitigate and respond to this growing problem [8]. For researchers in podiatry, changes to current online research practices are required to minimise the risk of non‐genuine participants to safeguard the data integrity and to report it transparently. The aim of this commentary is to raise awareness of the recently published practical strategies for researchers to ensure data reflects experiences of genuine participants and is not affected by non‐genuine and fraudulent responses.

## 2 Introduction

**FIGURE 1:**
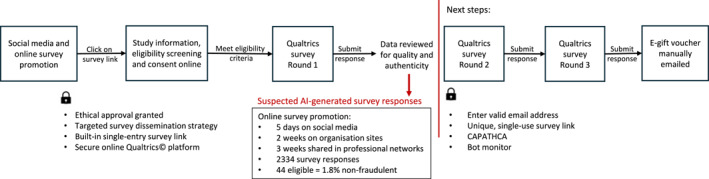
Flow diagram of our experience with AI‐trolling of online survey research.

In response to the threat of non‐genuine participation to data integrity in online research, a working group at the University of Nottingham has consolidated their personal experiences and strategies from previous studies [3, 4, 5, 6, 7] to develop guidance to help researchers ethically and effectively detect and deter non‐genuine responses [8]. To increase awareness of this evolving problem for researchers in podiatry and other health professions, the following sections summarise this published guidance. Our research team also presents an account of how artificial intelligence (AI)‐generated responses affected a recent online survey in podiatry, shown in Figure 1.

## 3 What to Look for?

Previous studies have outlined observed patterns that may prompt suspicion of non‐genuine participation in online research [3, 6, 7, 9], which include, but are not limited to, the following:

- Email addresses that were identically configured and repeated across entries (name.surname123@gmail.com), mismatched details, similar IP addresses, quick response to advert, concerns about payment, short emails in similar format across purportedly different participants, refusal to provide professional details, entries from countries outside the recruitment area, an abundance of participants claiming to be from highly underrepresented groups and demographic inconsistencies such as improbable combinations of age, location or profession.
- For surveys—unusual recruitment patterns such as a sudden surge in responses within hours of survey release, short or unrealistic completion times, contradicting responses, empty free text response boxes, high level of non‐responses, identical or highly patterned answers suggesting automated or inattentive responses.
- For interviews—unlimited participant availability, keenness for the process to happen quickly, cameras kept off during Zoom/Team interviews, short interview durations (< 30 min) compared with others, vague and confused answers, lack of elaboration through prompts, similarity between stories, inconsistencies in stories, and more frequent than usual enquiries about payments including the timing and type of voucher offered.

## 4 What to Do at Each Stage of Research?

The guidance states that strategies to minimise the impact of non‐genuine participation should be implemented as early as possible, ideally during grant writing and ethics application [8]. Key questions to ask when starting a project include: (1) What are the potential threats? (2) How would I find out? (3) What can I do before starting data collection? (4) What can I do during or after data collection? and (5) What can I do to confirm/ensure I am not excluding genuine participants? [8] Researchers are urged to embed explicit plans for verifying participant authenticity in study protocols and data‐management plans, and to consider implementing strategies at different research stages before, during and after data collection [8].

Practical steps to mitigate the risk of fraudulent and non‐genuine participants during data collection [6, 7, 8] include, but are not limited to, the following:

- Explain in participant information forms that data integrity checks will be performed to prevent fraudulent entries.
- Conduct small‐scale pilot testing to identify vulnerabilities in survey design.
- Allocate resources for data‐quality monitoring and participant verification technologies.
- Work with lived‐experience groups to design fair and acceptable verification processes for different populations.
- Recruit gradually rather than launching large‐scale adverts all at once, allowing time to identify suspicious trends, and avoid advertising on social media.
- Avoid the use of words, such as incentive or voucher, in public advertisements.
- Use CAPTCHA, unique login credentials, single‐use links and email/SMS verification to confirm human identity.
- Delay or place conditions on payments until participant validity is confirmed.

Technology, such as AI, used in online research continues to evolve with more sophisticated ways to undermine existing mitigation strategies. Currently published guidance is not prescriptive but is intended to support researchers in understanding key considerations and responding professionally to this ongoing issue [8]. Therefore, this guidance should be considered as a framework to improve understanding of potential risks and inform approaches, which may need to be context‐, country‐ and participant sample‐specific [8]. The guidance also cautions that verification must be proportionate [8]. Collecting personal identifiers or running video checks can protect data, but risk deterring genuine participants. Researchers and ethics committees must balance rigour with respect for privacy to ensure that genuine contributors feel trusted and valued [8].

## 5 How to Report Suspected Non‐Genuine Participation

Once data are, or have been collected, researchers should review the data and retain all suspicious entries rather than delete them, in order to maintain transparent documentation of suspected fraudulent activities [8]. Non‐genuine activity should be reported to ethics committees and, where appropriate, to funding bodies. Transparent decision‐making procedures should ensure that any exclusions are justified and auditable. When publishing, researchers should disclose the number of and reasons for participant exclusions, any data‐quality problems and describe the steps taken to address them [8].

## 6 Evolution of AI Technology

It is essential that safeguards are generated within the health research community worldwide to protect online research processes against external AI exploitation and potential corruption. At the same time, it is to be expected that external AI strategies and technologies will evolve to stay ahead of these safeguards, which will become obsolete as a result.

To be effective over time, guidance relating to the protection of research processes against non‐genuine participation should be based upon continually upgraded awareness of new external AI strategies as they emerge. Realistically, such awareness will be beyond the means of individual health research projects. It is therefore recommended that a centralised system is developed to monitor and track developing AI strategies. A key aim of such a process will be to make available to research teams clear, regularly updated non‐prescriptive guidance on measures they can use to identify and prevent potential non‐genuine research participation.

Such a process is ambitious in scope and will involve sophisticated technologies of its own, which may be costly. While guidance will continue to develop in response to, and possibly in anticipation of, AI evolution, it should consistently emphasise clear and transparent decision‐making and reporting procedures, and the use of combined strategies, rather than a single measure, to prevent or address non‐genuine participation.

## 7 Conclusion

By treating participant authenticity as a key and vulnerable component of research integrity, not an assumption, the profession can protect data quality in podiatry research to generate trustworthy patient‐centred evidence. Non‐genuine participation can infiltrate even the most well‐designed projects, potentially compromising the validity and reliability of the data, leading to incorrect conclusions and inappropriate recommendations for patient care. These findings suggest that researchers should anticipate risks from the earliest planning stages, embed verification steps into protocols and ethics and grant submissions and undertake vigilant data management and transparent reporting using a combined strategy approach.

## 8 Our Experience of Non‐Genuine Participants in Online Research

In September 2025, an online survey in podiatry‐led research was launched using the platform Qualtrics. Planned survey dissemination included promotion through podiatry clinical and social media networks in WA and sharing on online platforms of professional organisations and consumer involvement groups. Based on similar published studies, approximately 60 survey responses were expected for this first round of a three‐round e‐Delphi survey. During the first 3 weeks that the survey was open, 32 survey responses were completed. However, in the fourth week, the survey responses jumped rapidly to 2334 and on review the majority of these responses were suspected of being AI‐generated. AI‐generated responses included typical AI text styles (e.g., ‘effective management of common conditions—e.g., plantar fasciitis, arthritis and diabetic foot complications’.), sociodemographic data that did not seem plausible or realistic, fast completion times under 5 min, email accounts with a random sequence of numbers or letters (jpfhduyodl45677@gmail.com), using spam‐type email addresses (@atomicmail.io) and/or written responses that were in American English not Australian English. There were patterns where 10 responses in a row would follow the same formatting. Despite published guidance recommending the tracking of IP addresses to prevent multiple submissions and the inclusion of open‐ended questions requiring meaningful text input that automated bots struggle to replicate, we found that some survey responses suspected of being AI‐generated were originated from different Western Australian IP addresses, had correct WA postcodes and well written responses, which made it more difficult to identify non‐genuine participation.

The survey was immediately closed to stop further non‐genuine responses, and it was immediately reported to the UWA Human Research Ethics Committee as an adverse event and to the UWA IT Security and Finance as a potential fraud attempt targeting the research incentive of a $20 e‐gift voucher for those who complete all three online survey rounds. All AI‐generated survey responses were excluded from the study sample and were not sent the survey link to Round 2. Detection criteria to identify AI‐generated responses (from real human responses) using a structured process were developed by the research team. All survey responses were independently read and categorised by two investigators (MM and WG). After the independent data cleaning process was complete, the investigators discussed differences in their categories (likely to be human, suspicious and requiring review, likely to be AI‐generated) to obtain consensus regarding the AI‐generated responses to be removed from the data set. A third investigator (AB) assessed all categories, adjudicated cases of disagreement and determined the final category. To prevent further AI‐trolling in the subsequent survey rounds, Qualtrics security options were used to generate a unique‐link authentication that was sent to each participant providing survey access protection and the human authenticator CAPTCHA, additional mandatory email and name input screening and the bot detection monitor was implemented in the survey, which are verification measures designed to reduce spamming risk.

Impact on the study:

- Increased research team workload with unplanned hours spent verifying data, formalising our decision‐marking procedures for data cleaning, reporting discrepancies to ethics committees and rewriting methods in the study manuscript.
- Delay to research timeline in student project with no extra time within the constraint of the degree dissertation submission.
- Negative impact on student researcher morale.
- Loss of confidence in data integrity.

## Author Contributions

**Will Gurr:** data curation, investigation, formal analysis, writing – review and editing. **Michael McDougall:** data curation, investigation, formal analysis, writing – review and editing. **Abigail O’Brien:** data curation, investigation, formal analysis, writing – review and editing. **Kate Carter:** conceptualisation, methodology, project administration, supervision, writing – original draft, writing – review and editing.

## Funding

No external funding was received for this study. This study was conducted as part of the research component of the podiatry degree for students at UWA. Internal funding was allocated for study participants each to receive an e‐gift voucher valued at AUD $20.00 for those who successfully complete all three online survey rounds to mitigate high attrition rates in Delphi studies. The study aimed to recruit a maximum of 50 participants to generate sufficient data and as such a total budget of AUD $1000.00 was allocated to cover financial remuneration.

## Ethics Statement

Ethics approval was granted by the UWA Human Research Ethics Committee—reference: 2025/ET000427. All necessary permissions, from individuals and organisations including from social media page administrators and clinic staff, were obtained prior to posting recruitment advertisements for the study.

## Consent

The authors have nothing to report.

## Conflicts of Interest

The authors declare no conflicts of interest.

## Data Availability

The data that support the findings of this study are available from the corresponding author upon reasonable request.
